# Prevalence and Impact of Diagnosed and Undiagnosed Depression in the United States

**DOI:** 10.7759/cureus.28011

**Published:** 2022-08-14

**Authors:** Asim Handy, Rohan Mangal, Thor S Stead, R. Lane Coffee, Latha Ganti

**Affiliations:** 1 Biology, Detroit Country Day School, Detroit, USA; 2 Medicine, University of Miami Miller School of Medicine, Miami, USA; 3 Medicine, The Warren Alpert Medical School of Brown University, Providence, USA; 4 Internal Medicine, University of Central Florida, Orlando, USA; 5 Emergency Medicine, HCA Florida Ocala Hospital, Ocala, USA; 6 Emergency Medicine, Envision Physician Services, Plantation, USA; 7 Emergency Medicine, University of Central Florida College of Medicine, Orlando, USA

**Keywords:** clinical depression, undiagnosed depression, patient health questionnaire (phq-9), depression screening, depression

## Abstract

Background: The objectives of this study were: 1) estimate the impact and severity of both diagnosed and undiagnosed depression in the general US population 2) explore the demographics of depression based on its common symptoms 3) interpret Patient Health Questionnaire-9 (PHQ-9) scores to improve accuracy in identifying individuals with depression.

Methods: A random sample of 200 individuals was selected from a general US adult population to complete the Patient Health Questionnaire-9 (PHQ-9).

Results: Only 39.4% of respondents indicated that they had a formal diagnosis of depression. In contrast, 53% of participants have considered seeking help from a mental health professional. More importantly, 31.45% of respondents without a formal diagnosis had a PHQ-9 score of over 10 (moderate to severe depression).

Conclusions: The results indicate that undiagnosed depression exists in the US population and suggest that access to mental health services needs to expand across the nation.

## Introduction

Clinical depression is a mood disorder that causes a persistent feeling of sadness and loss of interest that can affect ones thinking and behavior. This condition can lead to a variety of physical and emotional problems and often requires long-term treatment [1]. In a normal brain, neurotransmitters successfully send chemical messages through electrical signals released and received by neuron synapses. Dopamine is responsible for regulating one’s motivation for reward [2]. When comparing the brain of a depressed patient, there are several abnormalities including brain shrinkage (atrophy), a loss of grey matter volume, and reduced functional activity in the hippocampus. There is also a depletion of the neurotransmitters in the central nervous system including serotonin, norepinephrine, or dopamine [3]. Personality traits such as low self-esteem, a family history of depression, childhood depression, or traumatic life events such as sexual or emotional abuse have also been associated with an increased risk for depression. In addition, individuals ages 18-25 years old, those who identify as two or more races, and women are more prone to depression [4]. Several forces interact to cause depression including faulty mood regulation by the brain, genetic vulnerability, and stimuli from traumatic events [5]. As the global coronavirus pandemic took place, depression cases in adults jumped from 8.5% before the pandemic to a staggering 27.8%. Research from the Boston University School of Public Health reveals that elevated depression rates have persisted into 2021, climbing to 32.8%, thus affecting one in three adults [6]. Depression typically occurs in multiple episodes, with signs and symptoms reoccurring often. Some of these symptoms include feelings of sadness and emptiness, frustration, sleep insomnia, lack of energy, reduced appetite and weight loss, anxiety, slow thinking, trouble concentrating, unexplained physical issues, and suicidal thoughts and actions [7]. Three of the more common methods used in depression treatment include cognitive behavioral therapy, which is a psychosocial intervention, interpersonal therapy, a form of therapy to improve interpersonal functioning, and psychodynamic therapy, an approach to facilitate a deeper understanding of one's emotions [8].

The Patient Health Questionnaire-9 (PHQ-9) is a self-administered questionnaire designed to screen, diagnose, and monitor the severity of depression. This assessment was derived from the original PHQ which aimed to address multiple health concerns including depression, panic, anxiety, etc. The PHQ-9 was developed by Spitzer, Williams, and Kroenke under a grant from Pfizer. This self-administered nine-question questionnaire assesses each criterion of depression in the Diagnostic and Statistical Manual of Mental Disorders (DSM) and is commonly administered at therapists’ or doctors’ offices to monitor and evaluates a patient’s mental health [9].

The objectives of this study were: 1) estimate the impact and severity of both diagnosed and undiagnosed depression in the general US population; 2) explore the demographics of depression based on its common symptoms; 3) interpret PHQ-9 scores to improve accuracy in identifying individuals with depression.

## Materials and methods

Design

A cross-sectional study was conducted in July of 2022. Data were collected through an anonymous questionnaire using an online survey platform. A convenience sample of 200 respondents included all adults in the general US population regardless of age, sex, race, or ethnicity. This study was given an exempt determination by our institutional review board (2022-578).

Data collection

The survey consisted of three parts: First, it inquired about participants' demographics including their age, gender, level of education, and income. Then the PHQ-9 assessment with a few follow-up questions regarding their diagnosis of Depression. For each of the nine questions on the Patient Health Questionnaire, the respondent was asked: “Over the last 2 weeks, how often have you been bothered by the following problems?” Each question had only four choices including “Not at all” (0 points), “Several days” (1 point), “More than half the days” (2 points), or “Nearly every day” (3 points). The scores from all nine questions were added together to compute a total score. The interpretation of the depression severity is as follows: 0-4 points indicates none-minimal depression, 5-9 indicates mild depression, 10-14 indicates moderate depression, 15-19 indicates moderate-severe depression and 20-27 indicates severe depression. A few follow-up questions were asked regarding whether the patient had been formally diagnosed with depression, thought of seeing a mental health professional, what prevents them from seeing a mental health professional, and a final open-response question allowing them to express any concerns about their depression that hadn’t previously been expressed.

Statistical analysis

Statistical analysis was performed using JMP version 16 (SAS Institute Inc., Cary, NC, USA). Parametric tests were used for normally distributed variables and non-parametric tests were used for skewed data. Chi-square testing was performed to assess correlation between a high PHQ-9 score and having a formal diagnosis of depression. A p-value of <0.05 was considered statistically significant.

## Results

The cohort comprised of 200 individuals with 57.7% females and 42.3% males. The age distribution was 14% between the ages of 18-24, 29% between the ages of 25-24, 32% between 35-44, 10% between 45-54, and 13% of adults over the ages of 54 years old. The median age was 38, with an interquartile range of 30 to 44, and a range from 18-77 years. 31% of respondents’ highest level of education was high school, 28% university, 23% postgraduate, 15% vocational technical college, and 2% middle school. The distribution of race in the US population was: 60% White, 17% Latino, 12% Black, 6% Asian, 3% multiracial, 1% Native American and 1% other. Race was approximately proportional to the US population with 63% of respondents indicating they were White, 12% Black, 9% Hispanic, 7% Asian, 3% multiracial, with 2% preferring not to say and 4% composed of various other ethnicities. The distribution of annual household income was <$25,00 (21%), $25,000-50,000 (21%), $50,000-75,000 (16%), and $75,000-100,000 (15%), and >$100,00 (27%). In terms of marital status, 33% of participants were single, 39% were married, and 16% were living with a partner. Forty-two percent of respondents had no children, 17% had one child, 20% had two children, 12% had three children, and 4% had four children.

How often do you experience little interest or pleasure in socializing?

Only 22.3% indicated that they never experience little interest or pleasure in socializing. 38.3% indicated that they experience this several days, 24.4% indicated more than half the days, and 14.9% every day.

How often do you feel down, depressed, or hopeless?

Only 27.9% indicated that they never feel down, depressed, or hopeless, a majority of 45.3% of respondents on several days, 18.9% on more than half of the days, and 8% feeling this nearly every day.

How often do you have trouble falling asleep, staying asleep, or sleeping?

This question had a relatively even distribution with 37.8% of respondents saying they have trouble falling asleep on several days, 20.4% saying not at all, 20.9% indicating more than half the days, and 20.9% indicating nearly every day.

How often do you feel tired or have little energy?

A majority of 47.3% felt tired/had little energy on several occasions, with 12.4% never/rarely feeling tired, 17.9% more than half of the days and 22.4% nearly every day.

How often do you have a poor appetite or overeating?

Most participants did not seem to have any severe appetite issues with 31.8% indicating no issues, 41.3% indicating some days, 15.4% saying more than half the days and 11.4% indicating nearly every day.

How often do you feel bad about yourself - or that you’re a failure or have let yourself or your family down?

31.8% of participants indicated that they never/rarely feel bad about themselves or a failure to their daily. 41.3% indicated that they feel this on several days, 15.4% on more than half of the days, and 11.4% nearly every day.

How often do you have trouble concentrating on things, such as reading?

37.3% of respondents indicated that they do not have trouble concentrating on tasks such as reading, 39.8% indicating several days, 14.4% on more than half of the days, and 8.4% nearly every day.

How often do you speak so slowly that other people could have noticed. Or the opposite - being so fidgety or restless that you have been moving around a lot more than usual?

55.2% of participants indicated that they do not move too slowly or restlessly. 24.9% indicated these actions on several days, 10.9% indicating more than half the days, and 9% indicating nearly every day.

How often do you have thoughts that you would be better off dead or of hurting yourself in some way?

Of the most severe question asked in the survey, a majority of 65.7% indicated no thoughts of being better off dead or hurting themselves, however 22.4% indicated that they have had these thoughts on several days, 8% on more than half of the days, and a dangerous 4% on nearly every day. This marks the final question of the PHQ-9 Survey.

Additional questions

Interestingly, only 39.4% of respondents have indicated that they have had a formal diagnosis of depression and 60.6% have not. In contrast, 53% of participants have considered seeking help from a mental health professional while 47% have not considered such a course of action. To account for this disparity, a follow-up question asked: “Has anything prevented you from seeking help from a mental health professional?” with the answer choices ranging from “I don’t need it”, “Too busy”, and “Can’t afford it” to “Afraid of stigma”, “Other”, or “None of the above”. The largest proportion of respondents claimed that they did not need it (32.1%) and the second highest proportion chose “None of the above” (23.9%). The final open-ended question had a wide range of responses. Some examples include: “I have been feeling very down and just tired all the time I can get more irritated very easily and I get frustrated I get annoyed easily to where I don’t want to deal with no one”, “I’ve been denied for social security 3 times already”. Notably, many responses called for the need to expand access to mental healthcare and drive down these costs. Some examples include: “Wish there were more access to free mental healthcare”, and “Mental health care should be more accessible in the USA”.

PHQ-9 scores

The distribution of PHQ-9 Scores was skewed with a mean score of 9.74 points. The minimum score reported was 0, with the 25th percentile being 5, the median was 9, the 75th percentile was 13.5, and the maximum score was 26.

**Figure 1:**
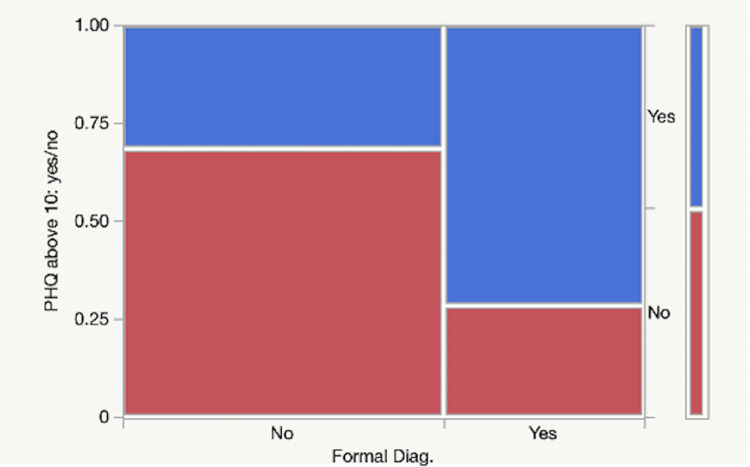
Prevalence of Patient Health Questionnaire-9 (PHQ-9) score > or = 10 in those who do and do not have a formal diagnosis of depression.

A statistical model was created to see whether the PHQ-9 score, age, or gender had an impact or not on whether the respondent had previously been diagnosed with depression. A high PHQ-9 score was significantly associated with a prior diagnosis of depression (P=0.0004). Age, gender, and race were not significant predictors. The adjusted R-Squared value is 0.184, meaning that 18.4% of the variation in a patient’s diagnoses can be explained by the model, indicating a solid statistical model. A separate column was generated analyzing those who had a PHQ-9 score of 10 or greater (moderate-severe depression). Almost half of the cohort (46.8%) screened positive. They were significantly more likely to have a formal diagnosis of depression when compared to those with lower scores (P<0.0001). More concerning was the 31.5% who did not have a formal diagnosis of depression but did have PHQ-9 scores >10 (Figure 1).

## Discussion

Our survey suggests that Americans continue to face numerous external and internal crises that can lead to negative thoughts, feelings, emotions, and eventually depression. The burdens and costs of having depression are the most severe of any disease (increased medical care use, lower quality of life, and decreased workplace productivity) [10]. Depression continues to be undiagnosed because its signs and symptoms are misunderstood. As noted, many people are reluctant to act towards seeing a mental health professional due to the stigma attached to this disorder. As of 2020, more than 21 million Americans, almost 8.4%, experienced at least one major depressive episode [11]. In fact, during the COVID-19 pandemic, depression and anxiety rates increased by more than 25% worldwide, impacting almost every stratum of society, including adolescents [12], college students [13], healthcare workers [14], and physicians [15]. Millions more will suffer from recurring depression in their lifetime. In addition, children are having an increased pressure for conformity in our modern society, especially in a rapidly evolving digital world. This danger imposes a risk of developing low self-esteem, one of the risk factors for depression. From this survey, it is evident that depression needs to be better identified. In the open-ended responses, many individuals noted that there is a shortage of mental health counseling in their community, that their insurance does not cover it, or they simply can’t access these facilities. However, nearly half of all respondents screened positive for moderate to severe depression.

Limitations

Some racial/ethnic groups such as Hispanics were not as well represented as they should have been. A majority of participants were in the two lower income brackets (less than $25,000 and $25,000-50,000 annual income respectively), with less participation from the middle and upper classes. Additionally, only adults over the age of 18 were surveyed, so the prevalence of undiagnosed depression in teenagers could not be measured. One other factor to note is that many individuals suffering from depression may have declined to respond meaningfully to the survey due to personal issues. One major limitation is much of the survey was multiple choice with only four answer choices. As a result, some respondents may have had to underestimate or overestimate their responses to choose an answer. In the future, more in-depth assessments and surveys within specific demographic groups, such as lesbian, gay, bisexual, transgender, queer and intersex (LGBTQI) may facilitate more targeted and effective treatment.

## Conclusions

Our data suggests depression is ongoing, and under-diagnosed. Increasing access to affordable care may be beneficial in diagnosis, treatment, and prevention. Overall, depression continues to be an ongoing problem in America and across the globe, and healthcare access and innovative solutions are imperative for improving our nation’s mental health and quality of life.
